# The transcriptome of the novel dinoflagellate *Oxyrrhis marina *(Alveolata: Dinophyceae): response to salinity examined by 454 sequencing

**DOI:** 10.1186/1471-2164-12-519

**Published:** 2011-10-20

**Authors:** Chris D Lowe, Luciane V Mello, Najma Samatar, Laura E Martin, David JS Montagnes, Phillip C Watts

**Affiliations:** 1 Department of Evolution, Ecology, and Behaviour, Institute of Integrative Biology, University of Liverpool, Biosciences Building, Crown Street, Liverpool, L69 7ZB, UK

## Abstract

**Background:**

The heterotrophic dinoflagellate *Oxyrrhis marina *is increasingly studied in experimental, ecological and evolutionary contexts. Its basal phylogenetic position within the dinoflagellates make *O. marina *useful for understanding the origin of numerous unusual features of the dinoflagellate lineage; its broad distribution has lent *O. marina *to the study of protist biogeography; and nutritive flexibility and eurytopy have made it a common lab rat for the investigation of physiological responses of marine heterotrophic flagellates. Nevertheless, genome-scale resources for *O. marina *are scarce. Here we present a 454-based transcriptome survey for this organism. In addition, we assess sequence read abundance, as a proxy for gene expression, in response to salinity, an environmental factor potentially important in determining *O. marina *spatial distributions.

**Results:**

Sequencing generated ~57 Mbp of data which assembled into 7, 398 contigs. Approximately 24% of contigs were nominally identified by BLAST. A further clustering of contigs (at ≥ 90% identity) revealed 164 transcript variant clusters, the largest of which (Phosphoribosylaminoimidazole-succinocarboxamide synthase) was composed of 28 variants displaying predominately synonymous variation. In a genomic context, a sample of 5 different genes were demonstrated to occur as tandem repeats, separated by short (~200-340 bp) inter-genic regions. For HSP90 several intergenic variants were detected suggesting a potentially complex genomic arrangement. In response to salinity, analysis of 454 read abundance highlighted 9 and 20 genes over or under expressed at 50 PSU, respectively. However, 454 read abundance and subsequent qPCR validation did not correlate well - suggesting that measures of gene expression via *ad hoc *analysis of sequence read abundance require careful interpretation.

**Conclusion:**

Here we indicate that tandem gene arrangements and the occurrence of multiple transcribed gene variants are common and indicate potentially complex genomic arrangements in *O. marina*. Comparison of the reported data set with existing *O. marina *and other dinoflagellates ESTs indicates little sequence overlap likely as a result of the relatively limited extent of genome scale sequence data currently available for the dinoflagellates. This is one of the first 454-based transcriptome surveys of an ancestral dinoflagellate taxon and will undoubtedly prove useful for future comparative studies aimed at reconstructing the origin of novel features of the dinoflagellates.

## Background

*Oxyrrhis marina *is a basal dinoflagellate taxon that has been extensively studied in both experimental and ecological contexts [1,2] and increasingly represents a target for studies of dinoflagellate evolution [3]. *Oxyrrhis marina *appears to have diverged early in the evolutionary branch leading to the dinoflagellate lineage, close to when the dinoflagellates diverged from the apicomplexans [4-6] and thus occupies a novel position within the alveolates (*i.e*. the ciliates, dinoflagellates, and apicomplexans). The alveolate lineages have each evolved a variety of unusual molecular and genomic features, the development of which has remained unclear in many cases [7]. The phylogenetic position of *O. marina*, as an intermediate lineage between the dinoflagellates and the apicomplexans, and the recognition that it possesses further unusual cytological and genetic features, make it a significant target for the study of evolutionary patterns and genome organisation within the alveolates [3,7-10].

Despite increasing scientific interest in *O. marina*, genetic and genomic data for this taxon remain relatively scarce (though see [8]). In part, this is because comparative genomic approaches are limited by the relatively large phylogenetic distances separating *O. marina *from other genetic/genomic-model protists (*e.g*. [4,5]). More generally, dinoflagellate genomes remain poorly-characterised due to several genomic characteristics. For example, dinoflagellates typically possess large genomes [7,11,12] that contain numerous genes arranged in repetitive tandem-arrays [13]; further, they have potentially complex transcriptomes composed of multiple transcript variants for many genes [14]. The occurrence of such traits in *O. marina *remains only partially characterised: the genome appears to be large [15], and a number of genes occur as multiple transcribed variants [16], but whether these genes are present as tandem-arrays has not been demonstrated.

While full genome sequences remain out of reach, next generation sequencing platforms nonetheless provide an efficient strategy to characterise transcriptomes, which can then be used to (1) quantify genomic features such as novel gene transcripts, alternative splicing, and levels of gene expression; and (2) uncover the molecular basis of adaptive traits in ecological model-organisms that lack reference genomes [17,18]. Thus, high throughput transcriptome sequencing represents a common starting point for large scale sequencing projects for a broad taxonomic range of organisms [18-20]. Indeed, several EST and transcriptome sequence datasets now exist for dinoflagellate species (*e.g*. [21,22], including an EST dataset for one strain of *O. marina *[8]. For *O. marina*, many components of its biology are well-characterised, and it is commonly employed as a model to parameterise ecological processes and trophic interactions (*e.g*. [23]). Additionally, as a result of its broad distribution and abundance in intertidal environments, *O. marina *is a useful model of the evolutionary and biogeographic processes that determine the distributions of free-living protists [24]. The wide distribution of *O. marina *is undoubtedly associated with an ability to tolerate a range of environments, notably variation in salinity, temperature, and pH [25]. Beneath this general pattern, however, is evidence for intra-specific variation in physiological tolerances; for example *O. marina *isolates display differing tolerances to environmental salinity, which potentially correlate with their occurrence in open water compared with intertidal habitats [26]. Crucially, the molecular basis of differences in physiological tolerances, and hence the mechanisms by which physiological adaptation potentially drives biogeographic patterns, are unknown. Identifying genes that respond to key parameters such as salinity stress represents the first step towards indentifying the basis of physiological differences between strains.

In this paper, we present the first 454-based transcriptome sequence data for *O. marina*, with the aim of (1) highlighting the occurrence of genomic features such as extensive gene transcript variants, tandemly-arrayed genes, and a gene complement that make it an important target for understanding genome evolution within the dinoflagellates. Moreover, (2) we assess the use of 454 read abundance to determine variation in gene expression in response to salinity stress, and thus we examine the potential molecular basis of salinity tolerance in this eurytopic flagellate. In doing so we provide a substantial dataset that increases the publically available DNA sequence resources for this highly unusual dinoflagellate species.

## Methods

### Cell culturing

To provide RNA for cDNA synthesis and subsequent 454 sequencing, monoclonal cultures of the *O. marina *isolate 44_PLY01 (source: Plymouth harbour, UK, 50.3632 N, -4.139 W; see [24]) were established in triplicate with media adjusted to 30 and 50 PSU (practical salinity units). Cultures were grown in modified Droop's S69 axenic growth medium [25] (see [27] for details) and were treated with gentamycin (50 μg ml^-1^) and penicillin/streptomycin solution (100 μg ml^-1^) to limit bacterial growth; absence of bacteria and fungi was confirmed by culturing small volumes of *O. marina *cultures in L1p, L1m, and L1pm test media (media recipes provided by The Provasoli-Guillard National Center for Culture of Marine Phytoplankton, Bigelow Laboratory, Maine, USA) and by visual inspection of DAPI stained culture aliquots using a UV-equipped inverted microscope. Cultures were maintained in the dark at 18°C and serially transferred to ensure exponential growth. Specific growth rate (μ, d^-1^) was calculated from daily estimates of cell density over 5 days (cell densities were estimated by counting 1 ml subsamples using a Sedgewick-Rafter chamber). Cultures were maintained at 30 and 50 PSU for > 10 generations and were harvested when 1 L flasks contained ~3.5 × 10^7 ^cells.

### RNA extraction and cDNA synthesis

Cells harvested from triplicate cultures were combined, and total RNA was extracted using an RNeasy extraction kit (Qiagen) following the manufacturer's standard protocol. RNA quantity and integrity was assessed using an Agilent Bioanalyser PicoRNA assay (Agilent technologies). cDNA template for sequencing was generated using the standard SMART cDNA synthesis protocol (Clontech). First strand cDNA was synthesised using Superscript II reverse transcriptase from ~0.75 μg total RNA. Sufficient template for sequencing (~5 μg) was generated by long-range PCR; briefly, cDNA was amplified in 90 μl PCR containing 1.5 μl first strand cDNA as template, 20 mM dNTPs, 12 μM SMART oligo, ~50 U Advantage 2 *Taq *polymerase (Clontech), and the manufacturer's standard PCR buffer; thermal cycling conditions were: 95°C for 1 min, followed by 18 cycles of [95°C 15s, 65°C 30s, 68°C 6 min]. Amplified DNA was purified using a standard column-based protocol (Qiagen).

### 454 sequencing and sequence assembly

Library construction and pyrosequencing was completed by the Centre for Genomic Research (CGR, http://www.liv.ac.uk/cgr/), University of Liverpool, UK on a 454 GS FLX system (Roche). Libraries created from 30 and 50 PSU salinity treatments were multiplex identified (MID-tagged) and then pooled for sequencing using 0.5 × GS454flx sequencing run. Sequencing reads were quality trimmed and adaptor sequences removed prior to assembly. Contig assembly was performed using Newbler (release 1.1.03.24, Roche), with overlap settings of 35 bp and 99% identity and default values for the remaining parameters. The overall assembly was performed using the combined sequence data for both salinity treatments, and differentially-expressed genes were identified subsequent to annotation.

### BLAST identity searches and sequence annotation

Sequence contigs were annotated using a priority BLAST search strategy [28]. Briefly, *O. marina *contigs were compared sequentially against the following databases: Uniprot (release 15.0), NCBI Nr (release 172.0), Pfam (release 24.0), and Smart (version 4.1); identity searches were conducted using BLASTX (for Uniprot, Pfam, Smart) and megaBLAST (BLASTN - for NCBI Nr) implementations of the standalone BLAST program. Initial BLAST parameters were word size 30 (BLASTN), 3 (BLASTX) and an e-value cut-off of 1e^-10^. After each search the contigs that were not identified at the selected e-value cut-off were queried against the subsequent database (see Table 1). This priority BLAST procedure was repeated for the remaining unidentified contigs at a less-stringent e-value cut-off (1e^-5^). Finally, contigs identified by BLAST (*i.e*. those identified at an e-value of 1e^-5 ^or better) were submitted to blast2GO [29] for GO category assignment [30].

**Table 1:** BLAST annotation summary

Database	e-value (1e^-10^)	% contigs ID'd	e-value (1e^-5^)	% contigs ID'd
All contigs (*n *= 6, 497)				
Uniprot	655	8.9	139	1.9
Nr	434	5.9	61	0.8
Pfam	118	1.6	343	4.7
Smart	8	0.1	61	0.8
All databases	1, 215	16.5	604	8.2
Large contigs (*n *= 901)				
Uniprot	322	35.7	79	8.8
Nr	71	7.9	130	14.4
Pfam	58	6.4	117	13.0
Smart	15	1.7	9	1.0
All databases	466	61.7	335	37.2

Two additional BLASTN searches (using parameters specified above) were conducted between the *O. marina *RNAseq dataset reported here, an existing genbank *O. marina *EST dataset, and the dinoflagellate EST genbank collection. The degree of similarity between *O. marina *datasets was further explored based on a CAP3 [31] assembly of the combined datasets (see following section).

### Analysis of expressed gene variants

The occurrence of gene variants/clusters was explored based on a CAP3 assembly [31] of 454 contigs; thus potential clusters/variants were identified as groups of contigs sharing ≥ 90% similarity. For each of the largest clusters indentified in this way, contributing contigs were aligned using Seqman (DNAstar Inc, USA) and obvious errors (e.g. homopolymer length variations) or mis-alignments edited manually. For CAP3 contigs that were identifiable and contained open reading frames > 200bp in length, dN/dS ratios were calculated using KaKs calculator [32]. A further assembly was performed, which included the *O. marina *CCMP1788 EST dataset. Prior to assembly the CCMP1788 EST data were screened for redundancy (≥ 99.5% identity, 50 bp minimum overlap), which reduced the data from 18, 024 sequences to 11, 024. Subsequently, the 454 RNAseq and EST datasets were assembled at ≥ 90%. Transcripts identified as shared between the datasets were subjected to BLASTX searches against the Genbank non-redundant protein database to infer identity.

### Tandem gene PCR and cloning

For 5 candidates the occurrence of a tandem gene organisation was assayed by PCR. Outward orientated primers were designed within 100 bp of the 3' and 5' ends of contigs. PCRs using Phusion polymerase (NEB, Cambridge) were conducted using genomic DNA as template and 3.0 pmol of each primer. In all cases PCR amplicon identity was confirmed by capillary sequencing using Bigdye v3.1 chemistry on an AB3130xl genetic analyser. In 4 of 5 cases, tandem spacer regions generated a mixed sequencing signal indicating the presence of multiple amplicons. For 2 candidates (alpha tubulin and HSP90), PCR products were cloned using the cloneJET blunt end ligation kit (Fermentas) and JM109 competent cells (Promega). For each gene, 24 transformants were sequenced in forward and reverse orientations.

### Differential transcript abundance

The relative abundances of sequence reads from the 2 RNAseq datasets were used to quantify the pattern of gene expression in *O. marina *exposed to 30 and 50 PSU. The representation of sequence reads from 30 and 50 PSU libraries for each 454 contig was normalised for total library size (*i.e*. the total number of reads contributing to the assembly), and statistically significant differences in relative abundance between salinity treatments were assessed using the pairwise Audic and Claverie, Fisher exact and Chi-squared tests implemented in the software package IDEG6 [33]. Statistical significance was taken at *α*≤ 0.05, following Bonferroni correction implemented by the IDEG6 software.

Gene expression predictions from sequence read abundances were validated by quantitative PCR (qPCR). PCR primers were designed against 16 contigs with a range of read abundances and 3 nominal control/housekeeping genes. Template for qPCR assays was the same as that used for 454 sequencing. Assays were performed following the manufacturers protocol in 15 μl reactions, containing 2x PowerSYBR green (Ambion, Inc, CA) and 3 pmol forward and reverse primer. Target and control primer efficiencies were estimated based on serial dilutions of cDNA template. All PCRs were performed in triplicate on an AB7500 quantitative PCR system. Relative normalised expression metrics were calculated based on ΔΔCT [34]. Consistency of control gene expression was assessed based on pairwise ΔΔCT comparisons between actin, alpha-tubulin, and beta-tubulin. 454 target read abundances were normalised to control gene abundance to allow direct comparison between 454 and qPCR expression metrics.

## Results

### Sequence output and assembly statistics

**Figure 1:**
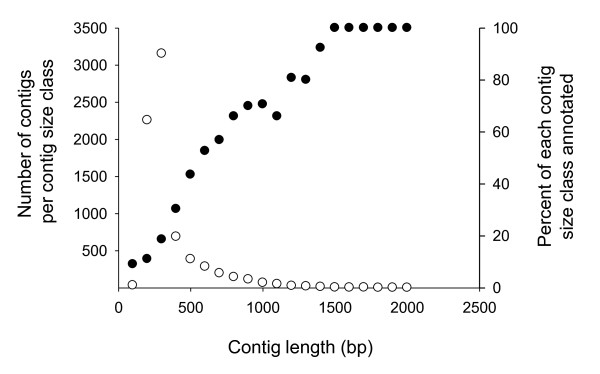
**A summary of BLAST annotation success for different contig size classes (filled circles), and contig size distribution (open circles)**.

Sequencing generated 299, 081 raw reads (totalling 57.2 Mbp) from the transcriptome of *O. marina *isolate 44PLY_01; following trimming to remove poor-quality and adaptor sequence, 238, 240 reads (median length 228 bp) were available for assembly. 194, 644 reads could be assembled, generating 7, 398 contigs, leaving 30, 869 singletons and 12, 727 reads that were removed as outliers. Median contig length and the mean read number per contig were 226 bp and 25.5, respectively. 2, 221 contigs were composed of 10 or more reads (10 - 2, 114 reads) and varied in length from 97-1, 938 bp - 901 contigs were greater than 500 bp long (between 4 and 1, 617 reads per contig; Figure 1). Short contigs (< 200bp) were typically represented by relatively small numbers of reads (median = 3). However, a small number of short contigs (*n *= 49) were highly represented in the sequence output (between 203 and 1, 909 reads per contig). Sequence reads are available via the NCBI sequence read archive (SRA accession SRA035395.1). Contig assemblies are included with this manuscript as Additional file 1, and summaries of the longest and most abundant contigs are included as Tables S1 and S2, respectively, in Additional file 2.

### Sequence Annotation

**Figure 2:**
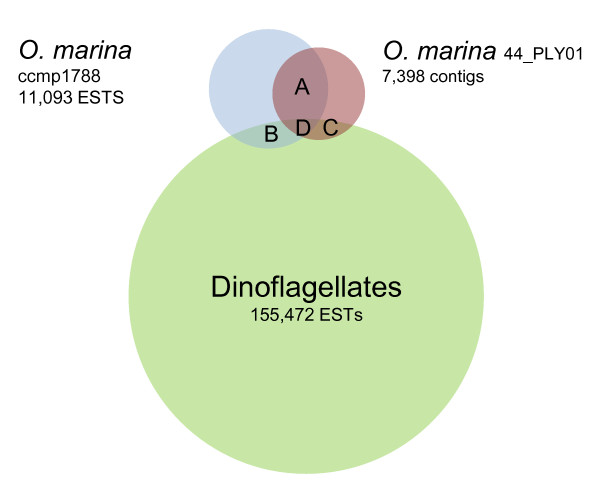
**A summary of reciprocal BLAST identity searches between *O. marina *sequence datasets and all EST sequences from Genbank**. The numbers of shared ESTs/contigs are: (A) 1410, (B) 372, (C) 161, (D) 495. Note that Venn diagram scaling is approximate.

Based on BLASTX and BLASTN identity searches against Uniprot, NCBI Nr, Pfam, and SMART databases we identified 16.5% of the *O. marina *contigs at the more stringent e-value cut-off (1e^-10^). A subsequent reduction in stringency (1e^-5^) allowed a further 8% of contigs to be assigned a putative function (thus, 76% of the *O. marina *contigs could not be identified, Table 1). Notably, ~98% of the large (> 500 bp) and all of the contigs greater than 1, 500 bp in length were identified by BLAST searches (Table 1, Figure 1), thus the relatively low annotation rate of the dataset stems from an inability to identify relatively short contigs (Figure 1, discussion). The overall degree of similarity between the *O. marina *RNAseq data presented here, an existing EST data set for *O. marina *(CCMP1788), and ESTs for the dinoflagellates as a whole was examined by further BLAST identity searches between these 3 datasets. Based on an e value of 1e^-10 ^and > 80% similarity the numbers of shared sequences are provided in Figure 2; *O. marina *data sets shared 1410 contigs/ESTs (~15%), and 495 contigs/ESTs were common to all three datasets.

### Transcriptome coverage, representation, and gene variants

**Figure 3:**
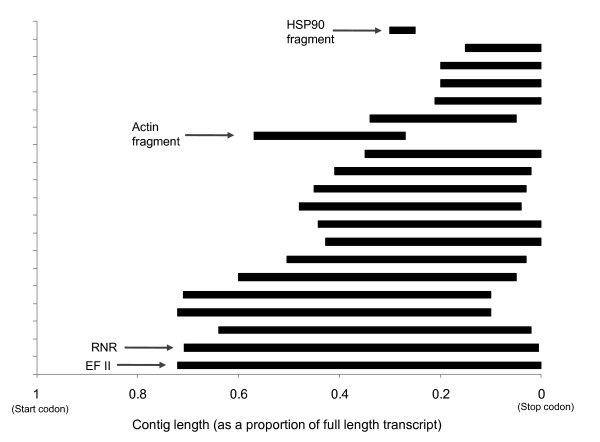
**The distribution of 20 contigs relative to their respective full length mRNA transcripts**. Transcript lengths were normalised to 1 (1 represents the position of the start codon and 0 the stop codon). The longest contigs (relative to total transcript length) were elongation factor 2 (EF II) and ribonucleoside-diphosphate reductase (RNR). Examples of 'contig fragments' of actin and HSP90 are indicated for reference.

**Figure 4:**
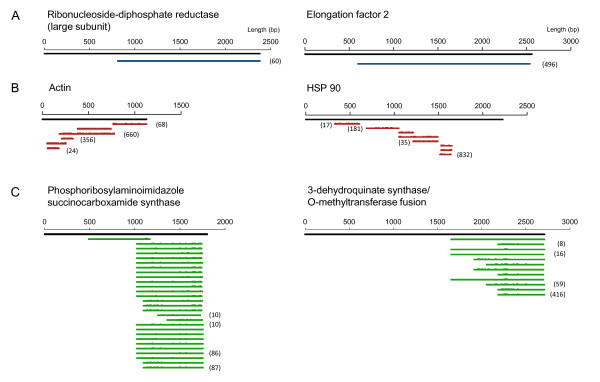
**Six examples of the different patterns of contig distribution in the *O. marina *454 RNAseq dataset**. (**A**, and blue lines) unigenes - unique, relatively long contigs; (**B**, and red lines) fragments - multiple short and partially overlapping contigs; (**C**, and green lines) gene variant clusters - multiple extensively overlapping contigs sharing ≥ 90%. Bracketed numbers indicate the numbers of reads contributing to the two most abundant and least abundant contigs for each alignment. Note that polymorphisms are indicated along contigs where the reference sequence was derived from *O. marina *44_PLY01.

To examine the relative coverage of transcripts by 454 sequencing, the length and aligned position of contigs relative to corresponding full length mRNA sequences was examined (Figure 3). For 20 contigs, full length mRNA sequences were available in Genbank for *O. marina*, or from relatively closely related taxa (*i.e*. basal dinoflagellates or *Perkinsus *spp). In all cases 454 contigs were truncated and no spliced leader sequences [9] were detected in the dataset. Relative contig length varied between 70 and 15% of the full mRNA transcript (Figure 3). The most complete identifiable contigs occurred for elongation factor 2 and ribonucleoside-diphosphate reductase large subunit (Figure 4a). Shorter contigs tended to cluster at the 3' end of mRNAs thus an overall 3' bias in coverage occurred (Figure 3).

**Figure 5:**
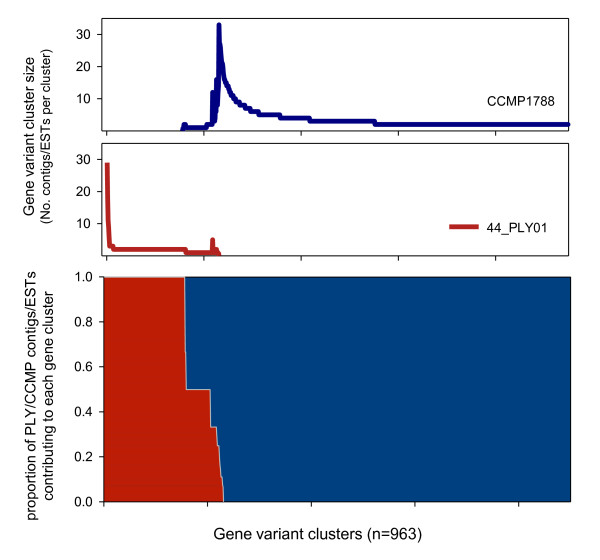
**A comparison of the 454 RNAseq (strain 44_PLY01) and EST (strain CCMP1788) sequence datasets**. Sequences were assembled using CAP3 (see methods). Bottom panel: the contribution (as a proportion of the total contigs/ESTS per gene cluster) of 44_PLY01 contigs (red) and CCMP1788 ESTs (blue) to each gene cluster. Note - columns that are entirely red represent gene clusters composed of 44_PLY01 contigs only; entirely blue columns represent gene clusters composed of CCMP1788 ESTs only. Middle and top panels: the size (*i.e*. the number of contributing contigs and/or ESTs) distribution of gene variant clusters in the 44_PLY01 (middle panel) and CCMP1788 (top panel) datasets.

In addition to single contigs some mRNA transcripts were represented as multiple non-overlapping fragments or as variable numbers of substantially overlapping fragments (Figure 4b, c). In the cases where multiple fragments occurred (*e.g*. HSP90 and Actin) one contig/fragment was typically highly abundant (Figure 4b). Manual checking of fragment alignments suggested that overlapping regions contained genuine polymorphisms, though the occurrence of multiple non-overlapping fragments is presumably an artefact of library preparation or sequencing (see discussion). The second arrangement, multiple highly overlapping contigs, appears to be a genuine biological signal (Figure 4c). To assess the prevalence, of these expressed gene variants, 454 contigs were further grouped/assembled using CAP3. This higher level assembly of contigs generated 164 gene clusters consisting of 2-28 contigs. In the majority of cases clusters consisted of truncated cDNAs bearing 3' UTR variants with < 100bp of coding sequence. However, for identified gene clusters, with > 200bp of coding sequence, variation was predominately synonymous, with d_N_/d_S _ratios between 0.33-0.023 (Table 2). For the most extensive gene variant clusters, contigs were predominantly highly overlapping and biased toward the 3' end of mRNAs (e.g. Figure 4c). A similar occurrence of gene variants was evident in the *O. marina *CCMP1788 EST dataset. An additional CAP3 assembly of the combined *O. marina *data highlighted a limited degree of overlap (74 CAP3 contigs contained sequences derived from both datasets) and ~750 gene variant clusters in the CCMP1788 ESTs (Figure 5). The most abundant gene cluster in CCMP1788 consisted of 33 variants, but was not identifiable by BLAST similarity searches against nr or nt databases. The largest gene variant cluster for which both strains possessed multiple gene variants was a type II rhodopsin gene.

**Table 2:** A summary of expressed gene variants and their synonymous/non-synonymous substitution rates

Gene	Copies	Alignment length (bp)	d_S_	d_N_	d_N_/d_S_
Phosphoribosylaminoimidazole-succinocarboxamide synthase	27	561	0.038 ± 0.011	0.0039 ± 0.002	0.123 ± 0.062
3-dehydroquinate synthase/O-methyltransferase fusion	12	408	0.0304 ± 0.012	0.008 ± 0.003	0.3294 ± 0.152
Heat shock protein 70	8	1353	0.0218 ± 0.006	0.0004 ± 0.004	0.0330 ± 0.0032
Calmodulin	6	447	0.0221 ± 0.0116	-	-
S-adenosylmethionine synthetase	5	1302	0.0294 ± 0.0082	0.0007 ± 0.0007	0.0226 ± > 0.0001
Chlorophyll a-b binding protein 25	2	213	0.122	0.009	0.076
Deoxyribonuclease-2-alpha	2	459	0.163	0.020	0.122
Glucose dehydrogenase	2	432	0.205	0.006	0.029
Heat shock protein 90	2	198	0.235	-	-
WD repeat-containing protein	2	190	0.170	-	-

### Tandem gene arrangements

**Figure 6:**
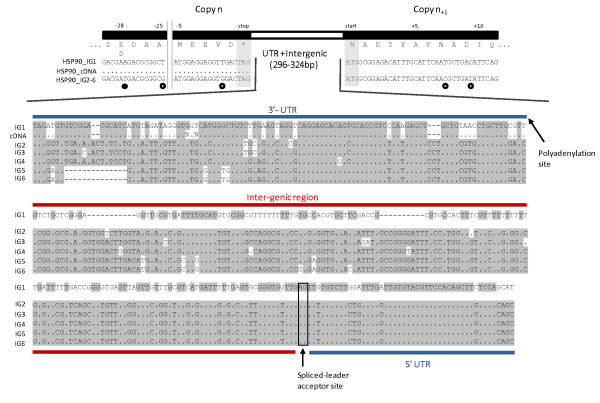
**Sequence alignment of 3' and 5' ends of the HSP90 gene and contiguous intergenic regions**. Coding regions have been translated to amino acid sequences to highlight the position of synonymous (open circles) and non-synonymous (filled circles) substitutions. Note a single non synonymous substitution occurred at amino acid position -28. The lengths of 3'/5' UTRs (blue) and intergenic regions (red) were inferred from comparison of genomic and cDNA sequences. IG1-6 refer to genomic sequence classes derived from PCR and cloning of the intergenic region.

The genomic context of variants was explored for 5 candidate genes (Table 3). In all cases, outward orientated primers designed against cDNA sequences generated PCR products confirming that genes were present as tandem repeated copies. Subsequent PCR and sequencing confirmed the length of intergenic regions (195-415bp). Cloning and sequencing of alpha tubulin and HSP90 PCR amplicons revealed the occurrence of a single 195bp intergenic region (IGR) for alpha tubulin and for HSP90, 6 variable IGRs (Figure 6). By percent identity HSP90 IGRs formed 2 groups (~82% identity); groups 1 and 2 consisted of 1 and 5 IGRs, respectively. Group 2 IGRs were also associated with an aspartic acid/glutamic acid substitution, occurring 28 residues from the stop codon (Figure 6). Five-prime ends of HSP90 and alpha tubulin cDNAs were confirmed to terminate in a generic dinoflagellate spliced leader sequence; the intergenic spacer/5' UTR boundary was inferred from alignment of cDNAs and genomic sequences. Five-prime UTRs were 45 and 77bp in HSP90 and alpha tubulin, respectively. In both cases spliced leader sequences were spliced at an 'AG' acceptor site (Figure 6).

**Table 3:** *Oxyrrhis marina *genes occurring in tandem repeats

Gene	Gene size (bp)	Tandem repeat? (Y - yes, N - no)	Intergenic region size (bp)
Beta tubulin	-	Y	-
Elongation factor 2	2499	Y	412
Rhodopsin	-	Y	-
Alpha tubulin	1362	Y	195
HSP90	2121	Y	321-340

### Gene content and functional annotation

A total of 571 contigs could be assigned to 1 or more GO categories (note that contigs may be assigned to several GO categories). GO annotation for biological processes (Level 3) highlighted the dominance of contigs associated with metabolic processes (46%), with fewer contigs involved with cellular organisation (*n *= 11%) and regulation (4%; Additional file 3, Figure S1). Similarly, contigs were assigned to a range of Level 3 Cellular Components, including intercellular components, membranes, organelles and protein- and ribonucleoprotein-complexes (Additional file 3, Figure S2).

**Figure 7:**
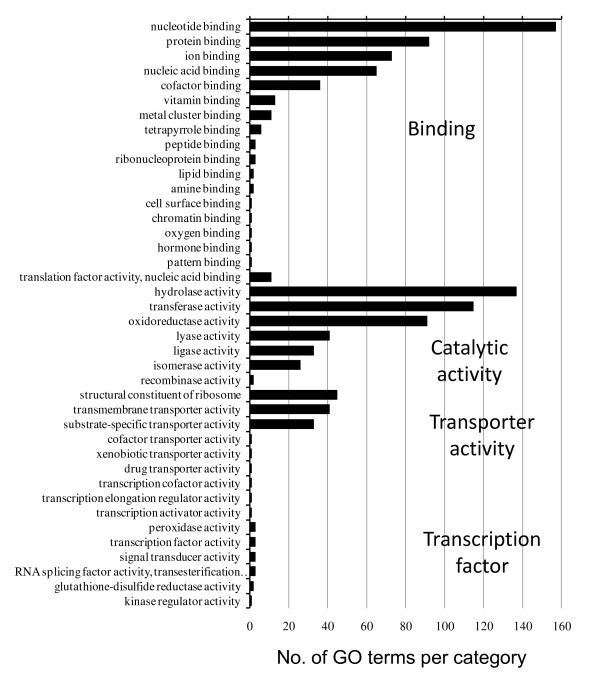
**A summary of functional annotation of 571 contigs based on GO classifications (GO level 3)**.

The functional distribution of the GO-assigned *O. marina *contigs is relatively typical of eukaryotes, representing a broad range of biological processes and cellular components. Notably 60-65 ribosomal proteins (Additional file 2, Table S3), out of an expected typical eukaryotic complement of 75-80 [35] were present. Figure 7 provides a summary of the distribution of molecular function (Level 3) categories and indicates that the majority of contigs are associated with binding processes (*n *= 468) and catalytic activity (*n *= 445). The most abundant transcripts connected with molecular binding processes include actin (*n *= 991 reads, protein binding), rho-associated protein kinase 2 (*n *= 381 reads, protein binding), V-type proton ATPase catalytic subunit A (*n *= 314 reads, nucleotide binding), serine/threonine-protein kinase (*n *= 150 reads, nucleotide binding), DNA replication licensing factor mcm7 (*n *= 123 reads, nucleotide binding), and nucleoside diphosphate kinase 7 (*n *= 119 reads, nucleotide binding). Abundant contigs were involved with catalysis include cathepsin B-like cysteine proteinase 6 (*n *= 1, 617 reads, hydrolase activity), aldehyde dehydrogenase (*n *= 460 reads, oxidoreductase activity), 2-nitropropane dioxygenase (*n *= 234 reads, oxidoreductase activity), ankyrin repeat-containing protein (*n *= 231 reads, hydrolase activity), calcium-dependent protein kinase (*n *= 226 reads, transferase activity), and 2-oxoisovalerate dehydrogenase subunit alpha (*n *= 202 reads, oxidoreductase activity).

A diverse range of genes associated with amino acid synthesis also occurred in the dataset. Based on the KEGG pathways database [36], 18 of the 22 amino acid biosynthesis, metabolism, and degradation pathways had partial representation in the *O. marina *dataset (Table 4) and 100 genes with discernable function within these pathways were detected (Additional file 4). Genes associated with the biosynthesis of 6 'essential' amino acids (valine, leucine and isoleucine, lysine, phenylalanine, tryptophan) were also present. Three of these amino acid/nitrogen metabolism genes - dihydrodipicolinate reductase 2 (associated with lysine synthesis), glutamine synthetase (associated with nitrogen glutamate synthesis), and ketol-acid reductoisomerase (associated with valine, leucine, and isoleucine biosynthesis) - are typically associated with plastids (Additional file 4, see discussion).

**Table 4:** Summary of contig assignments to KEGG pathways associated with amino acid biosynthesis and metabolism

KEGG ID	Kegg pathway	No. of contigs
00250	Alanine, aspartate and glutamate metabolism	8
00260	Glycine, serine and threonine metabolism	11
00270	Cysteine and methionine metabolism	8
00280	Valine, leucine and isoleucine degradation	12
00290	Valine, leucine and isoleucine biosynthesis	3
00300	Lysine biosynthesis	4
00310	Lysine degradation	6
00330	Arginine and proline metabolism	7
00340	Histidine metabolism	2
00350	Tyrosine metabolism	3
00360	Phenylalanine metabolism	4
00380	Tryptophan metabolism	7
00400	Phenylalanine, tyrosine and tryptophan biosynthesis	4
00410	Beta-Alanine metabolism	6
00430	Taurine and hypotaurine metabolism	2
00440	Phosphonate and phosphinate metabolism	0
00450	Selenoamino acid metabolism	3
00460	Cyanoamino acid metabolism	2
00471	D-Glutamine and D-glutamate metabolism	0
00472	D-Arginine and D-ornithine metabolism	0
00473	D-Alanine metabolism	0
00480	Glutathione metabolism	8

A complete list of assignments, and identifications are included in Additional file 4.

### Differential transcript abundance/gene expression in response to salinity

**Figure 8:**
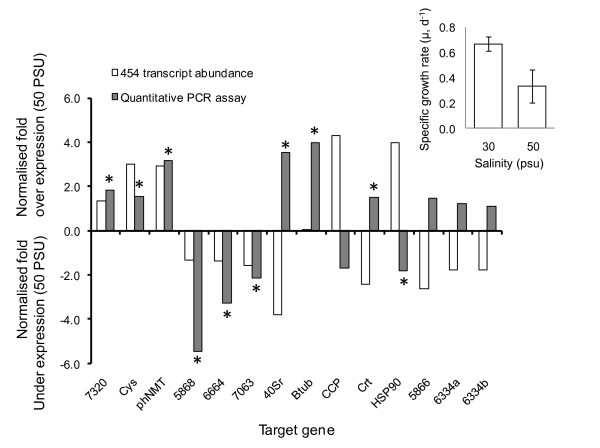
**454 read abundance and qPCR based estimates of relative expression in response to 2 salinity treatments**. (Main panel) comparison of relative gene expression (normalised to actin and alpha-tubulin) determined from qPCR assays and 454 read abundance. Target gene abbreviations are as follows: *Cys *- cysteine proteinase, *phNMT *- phosphoethanolamine N-methyltransferase, *40Sr *- 40S ribosomal protein, *Btub *- beta tubulin, *CCP *- cathepsin B cysteine proteinase, *Crt *- Calreticulin, *HSP90 *- heat shock protein 90 - numbers refer to contig ID where identifications were not made. * indicates significant over-expression based on qPCR assays (p < 0.05). (Inset) Mean specific growth rate responses of triplicate *O. marina *cultures grown at 30 and 50 PSU; error bars are standard deviations.

Growth rate of *O. marina *was significantly higher in the 30 PSU treatment (0.67 μ, d^-1 ^and 0.34 μ, d^-1 ^for 30 and 50 PSU, respectively: t_4 _= 5.72, p < 0.01; Figure 8). Based on an analysis of total read counts, 29 contigs were significantly differentially represented in salinity treatments (Tables 4 and 5). Nine and 20 contigs were over or under expressed (respectively) in the 50 PSU treatment. Of the ostensibly differentially expressed transcripts, 12 were nominally identified by BLAST identity (7 and 5 for 50 and 30 PSU libraries, respectively). Differences in expression varied markedly for transcripts over or under expressed at 50 PSU. For under expressed transcripts, differences ranged between 1.23 and 23 fold; total read counts varied between 26 and 2, 114 and were inversely proportional to fold difference (Table 5). For over expressed transcripts differences ranged between 1.3-1.8 fold, and total read counts varied between 210 and 1, 617 reads (Table 6). A large proportion of contigs (2, 285) were rare (< 12 reads) and occurred in only 1 of the 2 treatments (339 of which were identified). 1, 399 contigs were present in the 30 PSU treatment but absent in 50 PSU; 886 contigs were present at 50 PSU but absent at 30 PSU.

**Table 5:** Transcripts over expressed at 50 PSU

		Total	Normalised abundance		Pairwise differential		Closest BLAST
Contig	Length (bp)	reads	50 PSU	30 PSU	abundance (α 0.05)	ID (BLAST)	hit (GI No.)
05386	296	160	4.3	11.3		-	-
07320	629	210	14.3	8.1		-	-
00270	1938	496	32.6	19.9		elongation factor 2	3122059
06781	844	387	24.9	15.9	p < 0.001	deoxyribonuclease-2-alpha	3182984
07146	780	603	38.1	24.7		putative phosphoethanolamine N-methyltransferase 2	24212079
06493	249	738	44.5	32.8		glyceraldehyde-3-phosphate dehydrogenase	462139
06673	118	1158	69.7	51.5		heat shock protein 90	38885054
06337	220	893	53.7	39.8		cysteine proteinase precursor	32172429
06388	572	1617	94.2	74.4		cathepsin B-like cysteine proteinase	1169087

**Table 6:** Transcripts under expressed at 50 PSU

			Normalised abundance		Pairwise differential		closest BLAST
Contig	Length (bp)	total reads	50 PSU	30 PSU	abundance (α 0.05)	ID (BLAST)	hit (GI No.)
05236	109	26	0.1	2.3		-	-
06334	295	119	1.1	9.3		-	-
00224	199	35	0.4	2.9		-	-
06976	117	295	3.6	24.2		putative fumarate reductase	74581896
07063	294	148	3.1	10.4		-	-
06664	272	131	3.2	9.5		-	-
06325	275	442	11.3	31.6		-	-
05868	162	138	3.6	9.8		-	-
05866	175	279	7.1	19.1		-	-
04983	255	111	2.8	7.1	p < 0.001	-	-
06926	166	179	5.1	11.7		-	-
05228	243	192	6.4	12.6		-	-
05401	151	336	11.2	21.1		-	-
05956	131	691	26.2	42.8		-	-
06694	123	480	18.2	29.7		-	-
06660	221	632	26.2	37.4		calreticulin precursor	11131631
07447	146	725	30.5	42.5		-	-
05032	247	1058	46.8	60.3		conserved hypothetical protein	239877215
07041	200	1520	68.3	85.7		40S ribosomal protein S25	112253591
06775	258	2114	96.4	118.2		60S ribosomal protein L26-1	27735242
05386	296	160	4.3	11.3		-	-

Quantitative PCR validation assays were successful for 13 target genes (2 assays were excluded as a result of poor primer efficiency, and 2 assays were designed against 2 target gene to assess conformity). Target gene expression was normalised to 2 control genes (actin and alpha-tubulin, Figure 8). Six and 4 targets were identified as over or under expressed at 50 PSU, respectively, and greatest fold differences between treatments occurred for beta-tubulin, 40S ribosomal protein, and phosphoethanolamine N-methyltransferase (PhNMT) genes. Comparison of relative expression patterns estimated from qPCR and 454 read abundance indicated extensive discrepancies between the 2 approaches. In 6 out of 14 comparisons, the direction of expression differences was the same based on the 2 approaches. In the remaining 8 cases there were substantial differences in relative expression level estimates; for example, estimates of expression for HSP90 were 4.0 fold over expressed at 50 PSU versus 1.8 fold under expressed for 454 and qPCR based estimates, respectively (Figure 8).

## Discussion

### Oxyrrhis - an emerging genomic model

Recent interest in the genetic and genomic architecture of *O. marina *has informed the evolutionary history of a range of conspicuous dinoflagellate traits. For example, it is now clear that the RNA trans-splicing mechanism, seemingly ubiquitous within the dinoflagellates, also occurs within *O. marina *and the more distantly related *Perkinsus marinus *[37], suggesting that trans-splicing was established early in the ancestral lineage leading to the dinoflagellates. In contrast, studies of the mitochondrial genome indicate that the large but highly fragmented structure, again a feature of this taxon, is a more recent trait as it is common to the dinoflagellates and *O. marina *but probably not to *Perkinsus *[8]. Perhaps the most conspicuous dinoflagellate feature, the seeming massive genome sizes harboured by some species (up to 215 Gb [7]), also predates *O. marina *(a recent estimate places the genome size at ~50 Gbp [15]) but occurred after the divergence of *Oxyrrhis*/dinoflagellates from *Perkinsus*, in which the genome is of more typical proportion (~86Mb). Thus it is clear that *O. marina *is of increasing significance in the study of alveolate evolution.

Here we further indicate that tandem gene arrangements and abundant expressed gene variants are common in *O. marina*. EST surveys of several dinoflagellates have highlighted the occurrence of multiple transcripts coding for the same gene product [14,38], and detailed studies of specific genes have revealed complex gene arrangements and expressed gene variants in several species (e.g.[39,40]). In *O. marina*, previous study has shown abundant gene transcript variants for actin, HSP70, and rhodopsin (*e.g*. [3,16]). We indicate the same phenomenon here, with nominally 30 identified expressed genes (and ~130 anonymous truncated transcripts) present as up to 28 variants, for which the majority of nucleotide variation was synonymous. A comparison with existing ESTs indicates even more extensive gene variant clusters in *O. marina *CCMP1788. In both cases, a large number of variant clusters were not identifiable by BLAST searches against Genbank databases and no obvious functional class of genes appeared to dominant the most abundant variant clusters. For 44_PLY01 the largest gene cluster occurred for phosphoribosylaminoimidazole-succinocarboxamide synthase, a gene associated with purine metabolism [41], and the largest variant cluster in CCMP1788 coded for a type II rhodopsin gene. Notably, Slamovits et al. [16] have described ~50 variants encoding rhodopsin in strain CCMP1788; here we detected far fewer variants (2-4 rhodopsin contigs). This discrepancy may simply be a result of different methodologies. *Oxyrrhis marina *cultures in this study were grown in the dark, and given the likely role of rhodopsin in phototaxis [42], it would seem a potential that this treatment may reduce rhodopsin expression. Alternatively, differences in gene variant abundance may occur between strains. Whether structural or transcriptional differences exist at this level has yet to be examined, though global comparisons of the 44_PLY01 and CCMP1788 datasets using BLAST and CAP3 (Figures 2 and 5) both highlighted limited similarity (~15% of ESTs/contigs were common to both strains). We have previously documented extensive genetic diversity within *O. marina *[43] and strains CCMP1788 and 44_PLY01 occur within different *O. marina *clades (44_PLY01 and CCMP1788 occur within clades 1 and 2, respectively) based on sequence variation at 2 gene loci [1,24]. Whilst it is beyond the scope of the current study, it is likely that comparative assessments of gene/genome complement, arrangement, and structure at a range of phylogenetic between basal dinoflagellates will be highly informative. In particular, such comparative strategies will be useful to assess the rate of change of, for example, gene copy number at a key evolutionary juncture within the alveolates.

In addition to the occurrence of extensive expressed gene variants we have also shown that genes encoding transcribed variants occur as tandem repeated arrays in *O. marina*, an arrangement that has been demonstrated for a number of other dinoflagellate taxa [14]. The 5 genes examined here (Table 3) were each arrayed in tandem, separated by short intergenic regions. HSP90 occurs in several contexts, with 2 major variants of the intergenic region; notably however, based on 3' UTR sequence the variants detected in a genomic context did not tally with those present in the RNAseq dataset. Given that mRNA sequences for HSP90 were fragmented and incomplete at the 3' end it is most likely that the corresponding portion of transcripts were simply missing in the RNAseq data, though it is also possible that we have under sampled the existing variation for this gene. In contrast, only a single intergenic sequence was recovered for alpha tubulin, which did match the mRNA sequence. In both cases cDNAs were trans-spliced, a universal feature of dinoflagellate transcription [9], and the trans-splicing acceptor site corresponded to an 'AG' signal as noted in other dinoflagellates [14]. Of course, as a result of potential amplification biases associated with PCR detection, the actual diversity of intergenic regions is difficult to assess; nevertheless the occurrence of tandem gene repeats separated by different intergenic spacers suggests a number of potential genomic arrangements. Different intergenic spacers potentially indicate the occurrence of multiple tandem arrays at different genomic loci. Alternatively, individual arrays may be a complex arrangement of gene copies and heterogeneous intergenic spacers. Notably an *in situ *hybridisation based study of several genes in *O. marina *indicated 3, 4, and 5 genomic locations for actin, alpha tubulin, and HSP90 [15]. The precise structure and the extent of these tandem gene arrays remains to be investigated in *O. marina*; regardless, it is now increasingly clear that gene duplication is extensive in dinoflagellates more generally, and results in complex gene arrangements (e.g. [13,39]. Understanding the mechanisms promoting such expansions is an important focus for dinoflagellate genome biologists. A systematic survey of the arrangement of such duplicated genes will be informative and given the basal position of *Oxyrrhis *it will almost certainly prove valuable for establishing the likely origin of extensive duplication in the dinoflagellate lineage.

### The gene complement of *O. marina*

Analysis of the existing CCMP1788 EST dataset identified a range of *O. marina *genes indicative of significant evolutionary processes [3]. *Oxyrrhis marina *possesses genes such as proteorhodopsins that appear to have been laterally transferred from a bacterial origin [16] and a number of plastid genes, including ketol-acid reductoisomerase, carbonic anhydrase, and cysteine synthase, which suggests an evolutionary ancestry that included a chloroplast bearing cell [10]. In this study, we highlight the occurrence of a broad range of genes associated with amino acid synthetic and metabolic pathways, including genes which indicate the ability to synthesise 'essential' amino acids, a capacity not typical in heterotrophic protists. Molecular evidence for extensive biosynthetic capacities certainly supports previous study on the nutritional biochemistry of *O. marina*. A series of comprehensive studies of nutritional physiology by MR Droop and co-workers (*e.g*. [25,44]) highlighted that, in addition to phagotrophy, *O. marina *displayed a "plant-like" biochemistry including the ability to synthesise the full complement of amino-acids from ammonium or other simple nitrogen sources. While amino acid biosynthesis capability in heterotophic protists is exceptionally diverse, an absolute requirement for several amino acids is typical [45]. A broad range of transcripts indentified in this study were associated with amino acid metabolism and biosynthesis; based on the KEGG databases [36], 18 of the 22 amino acid biosynthesis pathways were represented by 100 454 contigs. The ability to undertake population growth on a fully synthetic medium with relatively simple absolute requirements (acetic acid or ethanol; valine, alanine; biotin; thiamine; vitamin B1s; ubiquinone; and a sterol [46]) and an exceptionally broad phagotrophic capacity (35-40 different prey items are documented as supporting *O. marina *population growth *in vitro *[27]) make *O. marina *exceptional. One mechanism by which *O. marina *may have gained its biosynthetic capacity is via an ancestral plastid or ancestral cyanobacterial endosymbiont [3]. The occurrence of plastid targeting signalling peptides and genes that are almost certainly plastid or cyanobacterial in origin (*e.g*. those coding for 1-deoxy-D-xylulose-5- phosphate reductoisomerase, haem, carbonic anhydrase, ketol-acid reductoisomerase, and dihydrodipicolinate reductase [3], and this study) are certainly strong support for such a mechanism.

More generally, based on GO and BLAST annotations a broad range of gene families and metabolic processes are nominally represented in the *O. marina *RNAseq library presented here. However, estimation of transcriptomic diversity, the comprehensiveness of the sequencing, and thus the likely gene complement of *O. marina *is difficult in the absence of a reference or close reference genome. Estimates of gene content based on genome size are possible; recent work by Hou and Lin [47] shows a strong non-linear correlation between genome size and protein-coding gene number across a broad range of eukaryotes. Hou and Lin [47] estimate total gene content of the largest dinoflagellate genomes to be on the order of 80-90, 000 genes comprising ~1% of the total genome. An estimated DNA content for *O. marina *of ~55.8 pg cell^-1 ^[15] places its genome within the dinoflagellate range (~50 Gbp) and suggests some ~70, 000 genes (assuming an average eukaryotic gene size of 1.3 Kbp [47]). Gene-content predictions of this magnitude are exceptionally high in comparison to other eukaryotes; however, as noted above, many genes in dinoflagellates occur in high copy numbers (up to 5, 000 gene copies in some cases, *e.g*. [40]); thus, it is possible that much of the 'gene space' in dinoflagellates is occupied by multi-copy genes and the total proteomic diversity is closer to that displayed by eukaryotes more generally [47].

The representation of conserved gene classes also provides an approximate indication of transcriptome coverage. In this study we detected 61 ribosomal protein coding transcripts of the 75-80 that are typical of most eukaryotes [35]; while contigs did not represent full transcripts and such a comparison can only give a crude estimate these figures suggest a representation in the region of 75% of the transcriptome. It should be noted however, that comparison of the RNAseq and EST datasets for *O. marina *potentially conflict with this estimate. Assuming strains are relatively similar (sequence divergence based on mitochondrial cytochrome oxidase I is ~2% [24]), the degree of overlap in transcriptome sequence datasets between strains was relatively small (~15%), potentially indicating a high degree of under-sequencing in both cases. Of course, strains might differ more than suspected, or biases in sequencing (*e.g*. truncation or fragmentation of transcripts) might reduce overlap between the datasets. In either case, it seems clear that comprehensive sampling of the *O. marina *transcriptome is likely to require a further substantial sequencing effort.

### Transcriptomic novelty and the problem of identification by identity

We have identified a number of interesting features of the *O. marina *transcriptome adding to previous descriptions of an unusual gene content in this organism. However, the majority of the sequences generated in this study were not identified by identity searches. This limited identification success, whilst partially accounted for by a 3' bias in this dataset (and thus a high representation of UTR sequence), is nevertheless diagnostic of a broader difficultly for genomic studies of dinoflagellates. While, the dinoflagellates are increasingly regarded as important targets for the study of genome evolution, large scale sequence resources are only relatively recently accumulating [21,22,48,49]. This poor sequence representation has an impact on the current use of such databases for sequence identification. For example, within the NCBI databases, EST datasets (totalling 155, 474 sequences) exist for only 21 dinoflagellate species, and the majority of ESTs (122, 235) are derived from just 5 species. Similarly, in a genomic context, only a handful of plastid genomes and genome sequence surveys exist for dinoflagellates and the majority of nucleotide sequences are environmental rDNAs. Consequently, identification of new sequences via database searches presents a significant challenge for dinoflagellate taxa.

In context, the relatively low annotation rate achieved in this study is, therefore, not surprising. EST projects on metazoa, with relatively close ancestry to many genomic model organisms, can yield high proportions of ESTs (*e.g*. > 95%) that are identified by reference to existing sequence databases (*e.g*. [50]). By contrast, only 1, 890 (16%) contigs were identified for *O. marina*, and less than 2% of transcripts matched to a single relatively closely related species, such as *Perkinsus marinus*. Comparably low rates of annotation have been reported for other dinoflagellate EST projects, with only 9% of the (~1, 400) ESTs isolated from *Alexandrium ostenfeldii *homologous to known proteins [48] and ~20% (of 6, 723) of ESTs from *Alexandrium tamerense *identified [21]. While ESTs from a number of other eukaryotic protist taxa, for example diatoms, do not appear to be so different from the protein and transcript data available in public databases, a typical annotation rate of ~50% of transcripts again highlights gaps in genomic information [48,51]. Most notably a recent EST project on *Perkinsus marinus *generated ~31, 000 EST sequences, clustered into ~8, 000 unique sequences of which 55% were identified [37]; possibly the higher annotation rate in this case is a result of the closer (relatively) phylogenetic affinity between *Perkinsus *and the Apicomplexa (a group that is well characterised by virtue of containing numerous parasites of humans and livestock). It is notable that only 145 *O. marina *transcripts produced significant identity to *P. marinus *sequences, and only 161 matches occurred between *O. marina *contigs and those from other dinoflagellate taxa. Whether, this is a genuine result of a high degree of novelty of the *O. marina *genome or a simple result of limited genomic data can only be confirmed by further genome scale sequencing, although inferences from phylogenetic analysis do suggest that *Oxyrrhis *represents a highly divergent and novel lineage [4].

### Identification of salinity tolerance mechanisms by differential gene expression

The application of next-generation sequencing technology to directly characterise transcript abundance is an increasingly used strategy for gene expression profiling [52-54]. The most precise strategies quantify either 5'or 3' (or both) cDNA fragments and thus overcome potential biases associated with sequence read length and incomplete reverse transcription [55]; but for species that lack genome references (for fragment mapping) this approach negates the generation of full or near full length coding sequences, which are typically a valuable output of transcriptome sequencing projects in the case of poorly characterised organisms. Our aim here was to determine whether a *de novo *transcript assembly can be used concurrently with an experiment to obtain an informative gene expression profile.

Comparisons of transcript abundance profiles for cells grown under 2 salinity treatments nominally identified differing gene expression patterns and in combination with growth rate estimates seemed to provide evidence for specific physiological responses and a tangible molecular mechanism. A higher maximum grow rate at 30 PSU, was concurrent with a relatively strong induction of ~20 transcripts at this salinity. Likewise, a reduced growth rate and modest induction of a different set of 8 genes occurred at 50 PSU. However, agreement between transcript abundance and qPCR gene expression estimates were relatively poor, both in terms of direction and magnitude, and in only 6 out of 14 assays were expression estimates similar. In a broader context, gene expression patterns derived via different methodologies (*e.g*. qPCR *vs*. microarray platforms) often do not strongly correlate, although there appears to be more concordance between qPCR and next-generation sequencing platforms than with microarrays (cf. [53,54]), which may relate to overall transcript abundance [52]. It is clear from a range of studies that some features of next generation sequencing protocols not specifically designed/targeted to quantify transcript abundance potentially generate significant biases in representation (e.g. [56]). From the study presented here for example, the representation of a number of gene transcripts in the *O. marina *RNAseq dataset by numerous non-overlapping fragments (with differing read abundances) is clearly problematic and is likely a result of either incomplete cDNA synthesis and/or a proportion of read assembly errors. Likewise, the occurrence of extensive expressed gene variants, seemingly common in most dinoflagellates has the potential to result in extensive discrepancy between sequence and qPCR bases approaches; particularly if qPCR assays co-amplify extensive gene variant families.

Accepting the above issues, those genes whose expression profiles were confirmed by qPCR did tentatively suggest a potential underlying salinity response. In 3 cases qPCR and 454 expression estimates identified genes as up regulated at 50 PSU; most notable was the up regulation of phosphoethanolamine N-methyltransferase - this enzyme is a component of a common pathway in plants that generates the osmoprotectant glycine betaine [57]. Thus, increased salinity appears to elicit a decrease in specific growth rate and tentatively a concurrent osmoregulatory response. Clearly such an inference is speculative and confirmation of the occurrence of this metabolic pathway in *O. marina *is required.

## Conclusion

We have generated some 7,398 cDNA sequence contigs for the basal dinoflagellate *O. marina*. BLAST searches identified ~14% of contigs; this relatively modest level of identification is likely due to *O. marina*'s unusual phylogenetic position and the limited sequence data for dinoflagellate taxa more generally. Nonetheless, we have identified a large number of transcripts associated with amino acid biosynthesis, and demonstrated the occurrence of extensive expressed gene variants and tandem gene arrangements; thus further highlighting the utility of next-generation sequencing platforms for generating *de novo *large scale sequence data to characterise non-genetic-model taxa. Additionally in this study, comparisons of relative read abundance of cells grown under differing osmotic stress nominally identified ~30 genes differentially regulated in response to salinity. While agreement between sequencing and qPCR based gene expression estimates was relatively poor; qPCR expression data tentatively identified candidate genes for further study of salinity tolerance in this taxon. In an evolutionary context, this is one of the first 454-based transcriptome surveys of an ancestral dinoflagellate taxon and will undoubtedly prove useful for future comparative studies aimed at reconstructing the origin of novel features of the dinokaryon. In an ecological context, these data highlight candidate genes for further research into potential adaptive mechanisms behind broad geographic distributions in eukaryotic microbes.

## Authors' contributions

PCW, DJSM, and CDL conceived and designed the study; CDL and LEM conducted culturing and molecular biology laboratory work; NS, LVM, CDL, and PCW undertook data analyses; all authors contributed to the manuscript. All authors read and approved the final manuscript.

## Supplementary Material

Additional file 1 ***Oxyrrhis marina *454 contigs**. Text file containing 7, 398 fasta-formatted sequences generated by Newbler (Roche 454) assembly. Click here for file

Additional file 2 **supplementary information for the BLAST identification of *Oxyrrhis marina *transcripts**. This file (.doc) contains summary tables of the longest contigs (Table S1) and the most abundant contigs (Table S2) and corresponding identifications (assigned by BLAST identity). Table S3 provides a summary of the ribosomal protein encoding genes present in the dataset. Click here for file

Additional file 3 **supplementary information for GO annotation of the *Oxyrrhis marina *transcripts**. This file (.doc) contains summaries of annotations for GO biological processes level 3 (Figure S1) and GO cellular component level 3 (Figure S2). Click here for file

Additional file 4 **supplementary information for contigs associated with KEGG amino acid synthesis pathways**. Data file (.csv) containing a summary of contigs associated with amino acid synthesis and a contig identification based on KEGG orthology. Click here for file

## References

[B1] Lowe CD Keeling PJ Martin LE Slamovits CH Watts PC Montagnes DJS Who is *Oxyrrhis marina? *Morphological and phylogenetic studies on an unusual dinoflagellate Journal of Plankton Research 2011 33 555 567 10.1093/plankt/fbq110

[B2] Montagnes DJS Lowe CD Roberts EC Breckels MN Boakes DE Davidson K Keeling PJ Slamovits CH Steinke M Yang Z Watts PC An introduction to the special issue: Oxyrrhis marina, a model organism? Journal of Plankton Research 2011 33 1 6 10.1093/plankt/fbq155

[B3] Slamovits CH Keeling PJ Contributions of *Oxyrrhis marina *to molecular biology, genomics and organelle evolution of dinoflagellates Journal of Plankton Research 2011 33 591 602 10.1093/plankt/fbq153

[B4] Saldarriaga JF McEwan ML Fast NM Taylor FJR Keeling PJ Multiple protein phylogenies show that *Oxyrrhis marina *and *Perkinsus marinus *are early branches of the dinoflagellate lineage Int J Syst Evol Microbiol 2003 53 355 365 10.1099/ijs.0.02328-0 12656195

[B5] Saldarriaga JF Taylor F Cavalier-Smith T Menden-Deuer S Keeling PJ Molecular data and the evolutionary history of dinoflagellates European Journal of Protistology 2004 40 85 111 10.1016/j.ejop.2003.11.003

[B6] Kato KH Moriyama A Itoh TJ Yamamoto M Horio T Huitorel P Dynamic changes in microtubule organization during division of the primitive dinoflagellate *Oxyrrhis marina* Biology of the Cell 2000 92 583 594 10.1016/S0248-4900(00)01106-0 11374437

[B7] Hackett JD Anderson DM Erdner DL Bhattacharya D dinoflagellates: a remarkable evolutionary experiment American Journal of Botany 2004 91 1523 1534 2004 10.3732/ajb.91.10.1523 21652307

[B8] Slamovits CH Saldarriaga JF Larocque A Keeling PJ The highly reduced and fragmented mitochondrial genome of the early-branching dinoflagellate *Oxyrrhis marina *shares characteristics with both apicomplexan and dinoflagellate mitochondrial genomes Journal of molecular biology 2007 372 356 68 10.1016/j.jmb.2007.06.085 17655860

[B9] Zhang H Hou Y Miranda L Campbell DA Sturm NR Gaasterland T Lin S Spliced leader RNA trans-splicing in dinoflagellates Proc Natl Acad Sci USA 2007 104 4618 4623 10.1073/pnas.0700258104 17360573 PMC1838650

[B10] Slamovits CH Keeling PJ Plastid-derived genes in the nonphotosynthetic alveolate *Oxyrrhis marina* Molecular Biology and Evolution 2008 25 1297 306 10.1093/molbev/msn075 18385218

[B11] LaJeunesse T Lambert G Andersen RA Coffroth MA Galbraith DW *Symbidiniun *(Pyrrhophyta) genome sizies (DNA content) are smallest among dinoflagellates Journal of Phycology 2005 41 880 886 10.1111/j.0022-3646.2005.04231.x

[B12] Veldhuis MJW Cucci TL Sieracki ME Cellular DNA content of marine phytoplankton using two new fluorochromes: taxonomic and ecological implications Journal of Phycology 1997 33 527 541 10.1111/j.0022-3646.1997.00527.x

[B13] Le QH Markovic P Hastings JW Jovine RVM Morse D Structure and organization of the peridinin-chlorophyll a-binding protein gene in *Gonyaulax polyedra* Molecular & General Genetics 1997 255 595 604 10.1007/s004380050533 9323363

[B14] Bachvaroff TR Place AR From stop to start: tandem gene arrangement, copy number and trans-splicing sites in the dinoflagellate *Amphidinium carterae* PLoS ONE 2008 3 e2929 10.1371/journal.pone.0002929 18698341 PMC2488372

[B15] Sano J Kato KH Localization and copy number of the protein-coding genes actin, alpha-tubulin, and HSP90 in the nucleus of a primitive dinoflagellate, *Oxyrrhis marina* Zoological Science 2009 26 745 53 10.2108/zsj.26.745 19877833

[B16] Slamovits C Okamoto N Burri L Erik RJ Keeling PJ A bacterial proteorhodopsin proton pump in marine eukaryotes Nature Communications 2011 2 183 6 21304512 10.1038/ncomms1188

[B17] Luikart G England PR Tallmon D Jordan S Taberlet P The power and promise of population genomics: from genotyping to genome typing Nat Rev Genet 2003 4 981 994 14631358 10.1038/nrg1226

[B18] Hudson ME Sequencing breakthroughs for genomic ecology and evolutionary biology Molecular Ecology Resources 2008 8 3 17 10.1111/j.1471-8286.2007.02019.x 21585713

[B19] Meyer E Aglyamova GV Wang S Buchanan-Carter J Abrego D Colbourne JK Willis BL Matz MV Sequencing and de novo analysis of a coral larval transcriptome using 454 GSFlx BMC Genomics 2009 10 219 10.1186/1471-2164-10-219 19435504 PMC2689275

[B20] Vera JC Wheat CW Fescemyer HW Frilander MJ Crawford DL Hanski I Marden JH Rapid transcriptome characterization for a nonmodel organism using 454 pyrosequencing Molecular Ecology 2008 17 1636 47 10.1111/j.1365-294X.2008.03666.x 18266620

[B21] Hackett JD Scheetz TE Yoon HS Soares MB Bonaldo MF Casavant TL Bhattacharya D Insights into a dinoflagellate genome through expressed sequence tag analysis BMC Genomics 2005 6 80 10.1186/1471-2164-6-80 15921535 PMC1173104

[B22] Moustafa A Evans AN Kulis DM Hackett JD Erdner DL Anderson DM Bhattacharya D Transcriptome profiling of a toxic dinoflagellate reveals a gene-rich protist and a potential impact on gene expression due to bacterial presence PLoS ONE 2010 5 e9688 10.1371/journal.pone.0009688 20300646 PMC2837391

[B23] Davidson K Sayegh F Montagnes DJS *Oxyrrhis marina*-based models as a tool to interpret protozoan population dynamics Journal of Plankton Research 2011 33 651 663 10.1093/plankt/fbq105

[B24] Lowe CD Montagnes DJS Martin LE Watts PC Patterns of genetic diversity in the marine heterotrophic flagellate *Oxyrrhis marina *(Alveolata: Dinophyceae) Protist 2010 161 212 21 10.1016/j.protis.2009.11.003 20034851

[B25] Droop MR Water-soluble factors in the nutrition of *Oxyrrhis marina* Journal of the Marine Biological Association of the United Kingdom 1959 38 605 620 10.1017/S0025315400007037

[B26] Lowe CD Day A Kemp SJ Montagnes DJS There are high levels of functional and genetic diversity in *Oxyrrhis marina* The Journal of Eukaryotic Microbiology 2005 52 250 7 10.1111/j.1550-7408.2005.00034.x 15927002

[B27] Lowe CD Martin LE Roberts EC Watts PC Wootton EC Montagnes DJS Collection, isolation and culturing strategies for *Oxyrrhis marina* Journal of Plankton Research 2011 33 569 578 10.1093/plankt/fbq161

[B28] Altschul SF Gish W Miller W Myers EW Lipman DJ Basic Local Alignment Search Tool Journal of Molecular Biology 1990 215 403 410 2231712 10.1016/S0022-2836(05)80360-2

[B29] Conesa A Götz S García-Gómez JM Terol J Talón M Robles M Blast2GO: a universal tool for annotation, visualization and analysis in functional genomics research Bioinformatics 2005 21 3674 6 10.1093/bioinformatics/bti610 16081474

[B30] The Gene Ontology Consortium The Gene Ontology in 2010: extensions and refinements Nucleic Acids Research 2010 38 D331 5 19920128 10.1093/nar/gkp1018 PMC2808930

[B31] Huang X Madan A CAP3: A DNA Sequence Assembly Program Genome Research 1999 9 868 877 10.1101/gr.9.9.868 10508846 PMC310812

[B32] Zhang Z Li J Zhao X-Q Wang J Wong GK Yu J KaKs_Calculator: calculating Ka and Ks through model selection and model averaging Genomics, Proteomics & Bioinformatics 2006 4 259 63 10.1016/S1672-0229(07)60007-2 17531802 PMC5054075

[B33] Romualdi C Bortoluzzi S D'Alessi F Danieli GA IDEG6: a web tool for detection of differentially expressed genes in multiple tag sampling experiments Physiological Genomics 2003 12 159 62 12429865 10.1152/physiolgenomics.00096.2002

[B34] Pfaffl MW A new mathematical model for relative quantification in real-time RT-PCR Nucleic Acids Research 2001 29 e45 10.1093/nar/29.9.e45 11328886 PMC55695

[B35] Marygold SJ Roote J Reuter G Lambertsson A Ashburner M Millburn GH Harrison PM Yu Z Kenmochi N Kaufman TC Leevers SJ Cook KR The ribosomal protein genes and Minute loci of *Drosophila melanogaster* Genome Biology 2007 8 R216 10.1186/gb-2007-8-10-r216 17927810 PMC2246290

[B36] Kanehisa M Goto S Kawashima S Okuno Y Hattori M The KEGG resource for deciphering the genome Nucleic Acids Research 2004 32 D277 80 10.1093/nar/gkh063 14681412 PMC308797

[B37] Joseph SJ Fernández-Robledo JA Gardner MJ El-Sayed NM Kuo CH Schott EJ Wang H Kissinger JC Vasta GR The Alveolate *Perkinsus marinus: *biological insights from EST gene discovery BMC Genomics 2010 11 228 10.1186/1471-2164-11-228 20374649 PMC2868825

[B38] Reichman JR Wilcox TP Vize PD PCP gene family in *Symbiodinium *from *Hippopus hippopus: *low levels of concerted evolution, isoform diversity, and spectral tuning of chromophores Molecular Biology and Evolution 2003 20 2143 54 10.1093/molbev/msg233 12949129

[B39] Zhang H Lin S Complex gene structure of the form II rubisco in the dinoflagellate *Prorocentrum minimum *(Dinophyceae) Journal of Phycology 2003 39 1160 1171 10.1111/j.0022-3646.2003.03-055.x

[B40] Bertomeu T Morse D Isolation of a dinoflagellate mitotic cyclin by functional complementation in yeast Biochemical and Biophysical Research Communications 2004 323 1172 83 10.1016/j.bbrc.2004.09.008 15451420

[B41] Ginder ND Binkowski DJ Fromm HJ Honzatko RB Nucleotide complexes of *Escherichia coli *phosphoribosylaminoimidazole succinocarboxamide synthetase The Journal of Biological Chemistry 2006 281 20680 8 10.1074/jbc.M602109200 16687397

[B42] Hartz AJ Sherr BF Sherr EB Photoresponse in the heterotrophic marine dinoflagellate *Oxyrrhis marina* The Journal of Eukaryotic Microbiology 2011 58 171 7 10.1111/j.1550-7408.2011.00529.x 21332875

[B43] Lowe CD Montagnes DJS Martin LE Watts PC High genetic diversity and fine-scale spatial structure in the marine flagellate *Oxyrrhis marina *(Dinophyceae) uncovered by microsatellite loci PLoS ONE 2010 5 e15557 10.1371/journal.pone.0015557 21203414 PMC3009739

[B44] Droop MR Pennock JF Terpenoid quinones and steroids in the nutrition of *Oxyrrhis marina* Journal of the Marine Biological Association of the United Kingdom 1971 51 455 470 10.1017/S002531540003191X

[B45] Hall RP TT C Nutrition and growth of protozoaresearch in protozoology 1967 1 Oxford: Pergamon Press Ltd 337 404

[B46] Droop MR Nutritional investigation of phagotrophic protozoa under axenic conditions Helgoland Marine Research 1970 277 272 277

[B47] Hou Y Lin S Distinct gene number-genome size relationships for eukaryotes and non-eukaryotes: gene content estimation for dinoflagellate genomes PLoS ONE 2009 4 e6978 10.1371/journal.pone.0006978 19750009 PMC2737104

[B48] Yang I John U Beszteri S Glöckner G Krock B Goesmann A Cembella AD Comparative gene expression in toxic versus non-toxic strains of the marine dinoflagellate *Alexandrium minutum* BMC Genomics 2010 11 248 10.1186/1471-2164-11-248 20403159 PMC2874808

[B49] Toulza E Shin M-S Blanc G Audic S Laabir M Collos Y Claverie JM Grzebyk D Gene expression in proliferating cells of the dinoflagellate *Alexandrium catenella *(Dinophyceae) Applied and Environmental Microbiology 2010 6 10.1128/AEM.02345-09 PMC2897438 20435767

[B50] Bai X Adams BJ Ciche TA Clifton S Gaugler R Hogenhout SA Spieth J Sternberg PW Wilson RK Grewal PS Transcriptomic analysis of the entomopathogenic nematode Heterorhabditis bacteriophora TTO1 BMC Genomics 2009 10 205 10.1186/1471-2164-10-205 19405965 PMC2686736

[B51] Maheswari U Mock T Armbrust EV Bowler C Update of the diatom EST database: a new tool for digital transcriptomics Nucleic acids research 2009 37 D1001 5 10.1093/nar/gkn905 19029140 PMC2686495

[B52] Wang Z Gerstein M Snyder M RNA-Seq: a revolutionary tool for transcriptomics Nature Reviews Genetics 2009 10 57 63 10.1038/nrg2484 19015660 PMC2949280

[B53] Marioni JC Mason CE Mane SM Stephens M Gilad Y RNA-seq: an assessment of technical reproducibility and comparison with gene expression arrays Genome Research 2008 18 1509 17 10.1101/gr.079558.108 18550803 PMC2527709

[B54] Wall PK Leebens-Mack J Chanderbali AS Barakat A Wolcott E Liang H Landherr L Tomsho LP Hu Y Carlson JE Ma H Schuster SC Soltis DE Soltis PS Altman N DePamphilis CW Comparison of next generation sequencing technologies for transcriptome characterization BMC Genomics 2009 10 347 10.1186/1471-2164-10-347 19646272 PMC2907694

[B55] Torres T Metta M Ottenwälder B Schlötterer C Gene expression profiling by massively parallel sequencing Genome Research 2008 1 172 177 10.1101/gr.6984908 PMC2134766 18032722

[B56] Duftner N Larkins-Ford J Legendre M Hofmann HA Efficacy of RNA amplification is dependent on sequence characteristics: implications for gene expression profiling using a cDNA microarray Genomics 2008 91 108 17 10.1016/j.ygeno.2007.09.004 18006269 PMC2258226

[B57] Mcneil SD Nuccio ML Ziemak MJ Hanson AD Enhanced synthesis of choline and glycine betaine in transgenic tobacco plants that overexpress phosphoethanolamine N-methyltransferase Proc Natl Acad Sci USA 2001 98 10001 10005 10.1073/pnas.171228998 11481443 PMC55567

