# Supplementary material for: The transcriptome of the novel dinoflagellate Oxyrrhis marina (Alveolata: Dinophyceae): response to salinity examined by 454 sequencing
Source: BMC Genomics. 2011 Oct 20;12:519. doi: 10.1186/1471-2164-12-519 (PMC3209475; doi:10.1186/1471-2164-12-519)
Supplement: Additional file 2 — supplementary information for the BLAST identification of Oxyrrhis marina transcripts. This file (.doc) contains summary tables of the longest contigs (Table S1) and the most abundant contigs (Table S2) and corresponding identifications (assigned by BLAST identity). Table S3 provides a summary of the ribosomal protein encoding genes present in the dataset. [file 1471-2164-12-519-S2.DOC]

**Table S1**. Longest transcripts.

| Contig | Length (bp) | No. reads | e-value | GI | Taxon | Nominal BLAST annotation |
| --- | --- | --- | --- | --- | --- | --- |
| 00270 | 1,938 | 496 | 0 | 3122059 | *Cryptosporidium parvum* | Elongation factor 2 |
| 05996 | 1,830 | 197 | 4e-34 | 75319668 | *Arabidopsis thaliana* | Calcium-dependent protein kinase 8 |
| 04950 | 1,828 | 90 | 7e-10 | 111991 | *Rattus norvegicus* | Resistance protein Mx1, interferon-regulated |
| 00449 | 1,813 | 94 | 5e-164 | 147744555 | *Homo sapiens* | Crooked neck-like protein 1 |
| 06573 | 1,788 | 126 | 3e-21 | 217979147 | *Methylocella silvestris* | Cobalamin synthesis protein P47K |
| 04795 | 1,697 | 49 | 4e-18 | 218511930 | *Debaryomyces hansenii* | Serine/threonine-protein kinase ATG1 |
| 06032 | 1,632 | 73 | 8e-48 | 303278316 | *Micromonas* sp. | Calcium-dependent cysteine proteinase |
| 05391^*^ | 1,623 | 44 | 2e-68 | 149278106- | *Pedobacter* sp. | Phytoene dehydrogenase |
| 06917 | 1,616 | 289 | 2e-19 | 239882191 | *Perkinsus marinus* | Hypothetical protein Pmar_PMAR024059 |
| 00589 | 1,607 | 62 | 9e-30 | 29839695 | *Arabidopsis thaliana* | Probable mitochondrial-processing peptidase subunit |
| 00606 | 1,596 | 60 | 0 | 417657 | *Caenorhabditis elegans* | Ribonucleoside-diphosphate reductase large subunit |
| 00643 | 1,591 | 108 | 5e-154 | 239890237 | *Perkinsus marinus* | Acyl-CoA dehydrogenase, putative |
| 00158 | 1,572 | 82 | 1e-19 | 74739702 | *Homo sapiens* | Probable E3 ubiquitin-protein ligase HERC2 |
| 00540 | 1,502 | 48 | 4e-69 | 20981695 | *Saccharomyces cerevisiae* | Fatty acid synthase subunit alpha |
| 04615 | 1,495 | 35 | 6e-65 | 48428640 | *Mus musculus* | Leucyl-tRNA synthetase, cytoplasmic |

^*^ Note/- contig 05391 is likely derived from a bacterial contaminant

**Table S2**. Most abundant transcripts.

| Contig | Length (bp) | No. reads | e-value | GI | Taxon | Nominal BLAST annotation |
| --- | --- | --- | --- | --- | --- | --- |
| 06775 | 258 | 2,114 | 1.0e-20 | 27735242 | *Arabidopsis thaliana* | 60S ribosomal protein L26-1 |
| 07116 | 116 | 1,909 |  | - |  | - |
| 06388 | 572 | 1,617 | 2.0e-29 | 1169087 | *Caenorhabditis elegans* | Cathepsin B-like cysteine proteinase 6 |
| 06491 | 120 | 1,524 |  | - | *-* | - |
| 07041 | 200 | 1,520 | 8.0e-12 | 112253591 | *Pfiesteria piscicida* | 40S ribosomal protein S25 |
| 07208 | 124 | 1,487 |  | - |  | - |
| 07355 | 105 | 1,487 |  | - |  | - |
| 06393 | 666 | 1,349 | 5.0e-16 | 224471826 | *Homo sapiens* | Serine/threonine-protein kinase/endoribonuclease |
| 05409 | 854 | 1,348 | 4.0e-43 | 74927078 | *Pandalus borealis* | Crustapain precursor |
| 07129 | 104 | 1,338 |  | - |  | - |
| 04773 | 349 | 1,323 |  | - | *Mus -* | - |
| 05385 | 361 | 1,217 | 2.0e-46 | 15638991 | *Schizosaccharomyces pombe* | 60S ribosomal protein L2 |
| 07351 | 209 | 1,216 | 1.0e-17 | 50403620 | *Rattus norvegicus* | 40S ribosomal protein S18 |
| 06673 | 118 | 1,158 | 8.0e-13 | 38885054 | *Oxyrrhis marina* | Heat shock protein 90 |
| 06518 | 230 | 1,113 | 3.0e-25 | 109895423 | *Anopheles gambiae* | 60S ribosomal protein L8 |

**Table S3. Ribosomal protein encoding genes**

| Contig | Length (bp) | No. reads | e-value | **GI** | BLAST annotation |
| --- | --- | --- | --- | --- | --- |
| 06985 | 539 | 25 | 3E-48 | 143462442 | 40S ribosomal protein S11 |
| 06621 | 762 | 131 | 3E-29 | 12229935 | 40S ribosomal protein S12 |
| 06965 | 211 | 6 | 1E-14 | 54039367 | 40S ribosomal protein S13 |
| 03887 | 197 | 2 | 2E-13 | 1173201 | 40S ribosomal protein S14 |
| 05635 | 527 | 8 | 9E-49 | 1173209 | 40S ribosomal protein S16 |
| 07203 | 391 | 68 | 7E-32 | 20141760 | 40S ribosomal protein S17 |
| 07351 | 209 | 1216 | 1E-17 | 50403620 | 40S ribosomal protein S18 |
| 07207 | 201 | 38 | 1E-22 | 157093577 | 40S ribosomal protein S19 |
| 00238 | 559 | 20 | 1E-63 | 730652 | 40S ribosomal protein S2 |
| 06289 | 203 | 2 | 3E-19 | 133883 | 40S ribosomal protein S20 |
| 04322 | 115 | 2 | 2E-12 | 464711 | 40S ribosomal protein S22 |
| 07128 | 537 | 59 | 4E-62 | 54039497 | 40S ribosomal protein S23 |
| 07041 | 200 | 1520 | 8E-12 | 112253591 | 40S ribosomal protein S25 |
| 04519 | 224 | 2 | 4E-15 | 157093575 | 40S ribosomal protein S28 |
| 07083 | 816 | 192 | 5E-77 | 75170994 | 40S ribosomal protein S3-3 |
| Table S3 continued | |  |  |  |  |
| 07335 | 367 | 35 | 2E-17 | 1350984 | 40S ribosomal protein S3a |
| 04909 | 181 | 5 | 2E-19 | 74844658 | 40S ribosomal protein S4 |
| 05430 | 701 | 18 | 6E-76 | 27734544 | 40S ribosomal protein S5-1 |
| 06931 | 225 | 10 | 5E-14 | 20139890 | 40S ribosomal protein S6 |
| 04650 | 204 | 24 | 2E-13 | 116256285 | 40S ribosomal protein S8 |
| 00608 | 616 | 71 | 1E-59 | 75309179 | 40S ribosomal protein S9-2 |
| 06604 | 284 | 161 | 4E-16 | 1172807 | 60S ribosomal protein L10 |
| 04766 | 222 | 7 | 3E-16 | 1709975 | 60S ribosomal protein L10a |
| 04345 | 241 | 3 | 4E-17 | 1350778 | 60S ribosomal protein L10E |
| 05543 | 389 | 18 | 6E-39 | 51704291 | 60S ribosomal protein L11 |
| 05815 | 763 | 29 | 7E-36 | 730528 | 60S ribosomal protein L13 |
| 06206 | 734 | 41 | 3E-66 | 6093871 | 60S ribosomal protein L15-1 |
| 00133 | 598 | 58 | 6E-43 | 730452 | 60S ribosomal protein L16-A |
| 06514 | 685 | 58 | 2E-58 | 75163854 | 60S ribosomal protein L18-3 |
| 00745 | 223 | 9 | 3E-11 | 237832139 | 60S ribosomal protein L18a |
| 05385 | 361 | 1217 | 2E-46 | 15638991 | 60S ribosomal protein L2 |
| Table S3 continued | |  |  |  |  |
| 07378 | 593 | 45 | 2E-44 | 2851508 | 60S ribosomal protein L21-1 |
| 04657 | 310 | 2 | 2E-12 | 118204 | 60S ribosomal protein L22 |
| 06312 | 173 | 6 | 2E-12 | 19884121 | 60S ribosomal protein L23 |
| 04964 | 180 | 5 | 6E-15 | 1710524 | 60S ribosomal protein L25 |
| 06775 | 258 | 2114 | 1E-20 | 27735242 | 60S ribosomal protein L26-1 |
| 02509 | 319 | 7 | 1E-17 | 400991 | 60S ribosomal protein L27 |
| 03639 | 211 | 2 | 8E-12 | 1350712 | 60S ribosomal protein L27a |
| 04893 | 483 | 10 | 1E-59 | 548770 | 60S ribosomal protein L3 |
| 06996 | 393 | 21 | 3E-38 | 17368245 | 60S ribosomal protein L30 |
| 06398 | 426 | 26 | 8E-31 | 51701835 | 60S ribosomal protein L31 |
| 05569 | 172 | 2 | 2E-13 | 21542436 | 60S ribosomal protein L3-2 |
| 05072 | 327 | 11 | 2E-19 | 17865566 | 60S ribosomal protein L36-3 |
| 05653 | 287 | 2 | 8E-13 | 74920963 | 60S ribosomal protein L38 |
| 00324 | 230 | 3 | 2E-16 | 51702811 | 60S ribosomal protein L39 |
| 01940 | 229 | 7 | 4E-15 | 1350746 | 60S ribosomal protein L40 |
| 07336 | 765 | 693 | 1E-42 | 17369604 | 60S ribosomal protein L4-1 |
| Table S3 continued | |  |  |  |  |
| 05231 | 216 | 3 | 3E-11 | 6831627 | 60S ribosomal protein L44 |
| 06695 | 322 | 164 | 2E-25 | 52783267 | 60S ribosomal protein L7 |
| 00008 | 316 | 12 | 4E-25 | 38503417 | 60S ribosomal protein L7-2 |
| 07324 | 867 | 161 | 3E-58 | 6174956 | 60S ribosomal protein L7a-1 |
| 06518 | 230 | 1113 | 3E-25 | 109895423 | 60S ribosomal protein L8 |
| 05744 | 638 | 31 | 5E-49 | 91207745 | 60S ribosomal protein L9 |
| 05978 | 157 | 54 | 2E-13 | 157093595 | 60S ribosomal proteins L8 and L2/40S ribosomal protein S15 fusion |
| 05895 | 240 | 95 | 5E-22 | 157093633 | ribosomal protein L17 |
| 04987 | 509 | 35 | 1E-61 | 157093621 | ribosomal protein L28e |
| 05649 | 224 | 6 | 1E-25 | 157093637 | ribosomal protein L35 |
