# Supplementary material for: The transcriptome of the novel dinoflagellate Oxyrrhis marina (Alveolata: Dinophyceae): response to salinity examined by 454 sequencing
Source: BMC Genomics. 2011 Oct 20;12:519. doi: 10.1186/1471-2164-12-519 (PMC3209475; doi:10.1186/1471-2164-12-519)
Supplement: Additional file 3 — supplementary information for GO annotation of the Oxyrrhis marina transcripts. This file (.doc) contains summaries of annotations for GO biological processes level 3 (Figure S1) and GO cellular component level 3 (Figure S2). [file 1471-2164-12-519-S3.DOC]

**Additional file 3, figure S1. A summary of functional annotation of 571 ESTs based on biological process GO classifications (GO level 3).**

Metabolic processes

Cellular processes

Localization

Response to stimulus

Regulation

**Additional file 3, figure S2. A summary of functional annotation of 571 ESTs based on cellular component GO classifications (GO level 3).**

Cell

Organelle

Macromolecular complex

Extracellular region
